# Cold Laser Micro-Machining of PDMS as an Encapsulation Layer for Soft Implantable Neural Interface

**DOI:** 10.3390/mi13091484

**Published:** 2022-09-07

**Authors:** Minjie Wang, Yuan Zhang, Jianxiong Bin, Lan Niu, Jing Zhang, Lusheng Liu, Aiping Wang, Jin Tao, Jingqiu Liang, Lihua Zhang, Xiaoyang Kang

**Affiliations:** 1 Laboratory for Neural Interface and Brain Computer Interface, Engineering Research Center of AI & Robotics, Ministry of Education, Shanghai Engineering Research Center of AI & Robotics, MOE Frontiers Center for Brain Science, State Key Laboratory of Medical Neurobiology, Institute of AI and Robotics, Academy for Engineering and Technology, Fudan University, Shanghai 200433, China; 2 Ji Hua Laboratory, Foshan 528200, China; 3 State Key Laboratory of Applied Optics, Changchun Institute of Optics, Fine Mechanics and Physics, Chinese Academy of Sciences, Changchun 130033, China; 4 Yiwu Research Institute of Fudan University, Chengbei Road, Yiwu 322000, China; 5 Research Center for Intelligent Sensing, Zhejiang Lab, Hangzhou 311100, China

**Keywords:** neural electrode, micro-machining, PDMS, excimer laser, electrochemical impedance, equivalent circuit model

## Abstract

PDMS (polydimethylsiloxane) is an important soft biocompatible material, which has various applications such as an implantable neural interface, a microfluidic chip, a wearable brain–computer interface, etc. However, the selective removal of the PDMS encapsulation layer is still a big challenge due to its chemical inertness and soft mechanical properties. Here, we use an excimer laser as a cold micro-machining tool for the precise removal of the PDMS encapsulation layer which can expose the electrode sites in an implantable neural interface. This study investigated and optimized the effect of excimer laser cutting parameters on the electrochemical impedance of a neural electrode by using orthogonal experiment design. Electrochemical impedance at the representative frequencies is discussed, which helps to construct the equivalent circuit model. Furthermore, the parameters of the equivalent circuit model are fitted, which reveals details about the electrochemical property of neural electrode using PDMS as an encapsulation layer. Our experimental findings suggest the promising application of excimer lasers in the micro-machining of implantable neural interface.

## 1. Introduction

Neural electrodes can record neural electrical activity or stimulate the neurons from the central and the peripheral nervous system, which has been applied to neuroscience research and clinical applications on different diseases, including spinal cord injury, stroke, sensory deficits, chronic pain, epileptic seizure, Parkinson’s disease, and functional recovery after neurotrauma [1,2,3,4,5]. Implantable neural electrode arrays are key interface devices for the development of intelligent brain–computer fusion systems, providing the possibility of continuous high-throughput information exchange.

Polydimethylsiloxane (PDMS) elastomers are inexpensive, corrosion-resistant, highly flexible and optically transparent above 220 nm (therefore compatible with many optical detection methods). Compared to glass and silicon, PDMS can be easily manufactured and bonded to other surfaces. The hardness and elasticity of PDMS is close to that of tissues [6,7] and does not irritate surrounding tissues due to its good biocompatibility [8]. With these ideal properties, PDMS is a good candidate for the manufacture of implantable flexible neural electrodes and has broad application prospects in the field of chip processing in biological laboratories [9].

Guo designed an integrated telescopic microelectrode array (isMEA) based on PDMS for neural and muscular surface interfaces [10]. As a compliant material with a mechanical impedance close to that of soft tissues, PDMS has good application value as a substrate material for neural interfaces. Using PDMS as a soft material, Kim prepared various types of planar neural electrodes for recording extracellular local field potentials or stimulating plexuses as contact electrodes [11]. Delivopoulos proposed a compliant neural interface based on PDMS that can simultaneously record bladder afferents from multiple nerves [12]. PDMS film has been proven to act as an excellent barrier to implantable microelectrodes or as high-quality dielectrics with minimal resistive power loss [13,14]. As an encapsulation layer for implantable neural interface, PDMS can increase the flexibility required for the application and minimize tissue response [15]. Joint research on other materials of PDMS provides a new idea for the further development of implantable neural interfaces [16]. A soft neural implant fully based on PDMS as the electronic dura mater was also proposed for spinal cord injury rehabilitation, which can narrow the mechanical mismatch gap between soft neural tissues and neural implants [17].

PDMS is an important material for biological application including neural interface [18,19,20,21,22,23,24], but it is not easy to accurately remove the PDMS encapsulation layer due to its chemical inertness and mechanical softness. Of course, PDMS film can be easily mechanically punched with relatively low spatial resolution. For higher resolution, PDMS film can also be micro-machined by reactive ion etching (RIE) with a maximum etch rate of 20 μm/h [25]. However, the RIE of PDMS usually requires extra mask on PDMS and the etch rate is relatively slow. Recently, the laser-processing technology of PDMS has emerged, which can enable fast and maskless fabrication. Many researchers use laser ablation technology, which works through a linear light absorption mechanism of light intensity to perform graphic processing on metal, conductor, and polymer surfaces [26,27]. PDMS has been modified and micromachined by femtosecond laser pulses [28,29,30,31,32,33], which can process most polymer and metal materials without regard to the over etching of different layers. Wolfe et al. used high-intensity light from a Ti: sapphire femtosecond pulse laser to create patterns on PDMS surface for soft lithography and microfluidics [34]. The bas-relief pattern required in these applications is usually fabricated by casting the PDMS on a complementary bas-relief pattern in a photoresist and then manufactured by lithography. These rough, concave features produced by this process are useful in applications that require a large surface area-to-volume ratio, and are not suitable for planar machining. Femtosecond laser processing provides widths as low as 1 μm for 3D channels [35], which is as low in size as soft lithography. Rather than manufacturing polymer channels, femtosecond lasers are more suitable for machining channels in harder materials such as quartz [36].

PDMS can also be micromachined by infrared and near-infrared laser [37,38,39], which may apply additional heat on the PDMS layer. Lin Qi reported a method of using an infrared commercial laser engraving machine to write surface wrinkles with the desired pattern geometry on PDMS [37]. This method is easy to operate, fast, and low-cost, but the infrared laser creates wrinkles and cracks on the surface of the PDMS, which does not meet the requirements for precise processing of the PDMS on the surface of the neural electrodes.

The types of laser sources available are diverse and the principles are different, so the heat-related side effects vary greatly in the laser processing of PDMS. Despite of the pulse width of the laser, the shorter wavelength can induce less heat during the fabrication process. PDMS can be micromachined by ultraviolet laser [40,41,42], which induces less heat side effect on PDMS. In particular, PDMS are sensitive to ultraviolet light below 230 nm; hence, excimer lasers that, in the lower band of the ultraviolet band, can be used for precise processing without the need for sensitizers [43,44,45]. Using excimer lasers as a cold micromachining tool, it is promising to selectively remove the PDMS encapsulation layer without damaging the other materials of the devices.

In order to find the optimal laser micro-processing parameters, here we performed extensive experiments to process the PDMS on the neural electrode surface without damaging the surface metal of the electrode. Firstly, we prepared PDMS-encapsulated neural electrodes. Then, excimer laser cutting parameters were designed and selected to precisely remove the PDMS encapsulation layer which can expose the electrode sites in an implantable neural interface. The electrochemical impedance spectroscopy (EIS) was analyzed to construct the equivalent circuit models, which can contribute to revealing the details of the neural electrode.

## 2. Materials and Methods

### 2.1. Neural Electrode

**Figure 1:**
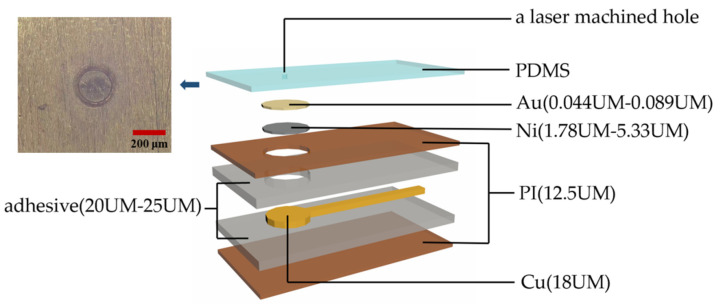
Soft neural electrode based on FPC technology and PDMS encapsulation layer. The PDMS layer serves as an intermediate layer which can reduce the mechanical mismatch between the flexible polyimide layer and neural tissue.

The flexible printed circuit (FPC) board is lightweight, small in size, and has good insulation properties, sealing properties, and radiation resistance. The properties that can be dynamically bent, curled, and folded give it a good advantage in the preparation of invasive neural interface devices. Here, we used the single-layer flexible circuit board, and the structure is shown in Figure 1. The upper and lower sides of the Cu (thickness 18 μm) and polyimide (thickness 12.5 μm) are glued together with adhesive (thickness 20 μm–25 μm). The flexible circuit board is then plated with nickel (thickness is 1.78 μm–5.33 μm) and Au (thickness is 0.044 μm–0.089 μm).

The material of the encapsulation layer used here was 184 Silicone Elastomer Kit of Dow Corning (DOWSIL). First, basic monomer A and curing agent B were mixed thoroughly in a 10:1 weight ratio. Then, the mixture was put into a vacuum dryer to remove the bubbles created in the mixing process by using a low-pressure method. The liquid PDMS was applied on 4-inch glass wafer at a spinning speed of 4000 r/min and cured in a drying oven at 80 degrees for 3 h, which acted as a substrate to secure the adhesion of the electrodes to facilitate subsequent processing. Another part of liquid PDMS was applied evenly to the neural electrode and then suspended vertically for two hours. Finally, the wafer and the electrodes were baked in a drying oven at 80 degrees for at least 3 h in order to fully cure. Here, the PDMS encapsulation layer acted as a soft mechanical layer for the FPC-based flexible neural electrode, which can reduce the mechanical mismatch between the neural tissue and flexible neural electrode. This strategy can also be applied to rigid neural electrodes such as Si probes. The flexible circuit board (FPC) technology we used is flexible but not soft because the Young’s modulus of polyimide is in the order of GPa (10^9^ Pa). However, PDMS is so intrinsically soft that its Young’s modulus is in the order of MPa (10^6^ Pa). Therefore, by encapsulating the flexible electrode with a soft PDMS layer, we want to give the softness to the flexible electrode. This approach has the potential to narrow the mechanical mismatch gap between soft neural tissues and flexible neural implants.

Shown in Figure 1, we used an excimer laser as a cold micro-machining tool for the precise removal of the PDMS encapsulation layer, which can expose the electrode sites in an implantable neural interface. The different laser parameters and their combinations determine whether the PDMS encapsulation layer can be accurately removed and whether it causes damage to the neural electrodes below. If the laser pulse energy used is too high or the number of hits is too high, the laser will not only penetrate the PDMS encapsulation layer but also damage the Au and even the Ni and Cu of the electrode, resulting in a decrease in the performance of the electrode, including electrochemical impedance and biocompatibility. The purpose of the optimization is to find the exact state in which PDMS encapsulation layer can be removed completely without damaging the neural electrodes, that is, the optimal combination of laser processing parameters.

### 2.2. Excimer Laser

An excimer pulse laser with a wavelength of 193 nm (filled with an ArF premix gas, Optec Micro Master device) was used as a laser source in our study to remove PDMS encapsulation layer which can expose the electrode sites. Excimer lasers output ultraviolet light, and PDMS can absorb ultraviolet light below 220 nm. The ArF lasers emit 6.4 eV photons, which are much higher than the bond energy in $\mathrm{Si}-{\mathrm{CH}}_{3}$ (3.2 eV) and Si–O (4.6 eV). Therefore, PDMS can be decomposed by ArF laser. Because the 193 nm laser can be fully absorbed by PDMS, it can be processed precisely without thermal effects compared with other laser processing methods [46].

The cutting parameters of excimer laser we studied included laser pulse energy, number of processes, and pulse repetition rate. The laser emitted by the laser source was trimmed through the template in size and shape to form the desired spot. The PDMS encapsulation layer was micro-machined one by one with the excimer laser to form the electrode site after aligning the laser beam to the specific position on the neural device. It is possible that the excimer laser can remove PDMS with sizes of tens of microns. However, this approach needs a higher precision displacement positioning platform to align the beam and electrode. Here, we studied and compared the processing of the 200 μm diameter circular template and the 200 * 450 μm rectangular template.

### 2.3. Orthogonal Experimental Design

Firstly, we conducted a series of experiments which covered a range of laser pulse energy 0.5 mJ–8 mJ, laser processing times 1 times–1500 times, and repetition rate 1 Hz–500 Hz. Parameters that are too small or too large, and the combinations between them, do not yield satisfactory experimental results. As is shown in Table 1, when using low laser pulse energy and small number, the impedance is high because the PDMS on the electrode surface is not penetrated. When using low pulse energy and large number, the PDMS cutting edges are not neat. When using high laser pulse energy, the impedance is too low because the laser not only penetrates the PDMS but also damages the Au on the electrodes. With minimal processing time and energy consumption, we wanted to find just the right state to penetrate the PDMS without damaging the electrodes.

After determining the parameter range, we performed a combinatorial experiment on these parameters. One optimization method is the method of factorial, that is, each level of each factor is tested at each level of each other factor. This approach increases the possibility of finding an exact optimal value for each factor, but it is too cumbersome. Another method, such as the orthogonal experimental design created by Taguchi, is based on the fractional principle of factorial design, using an orthogonal table derived from combination theory. The optimum levels for many different parameters can be simultaneously discovered, which can greatly reduce the time and cost [47,48]. To find the optimal excimer laser cutting parameters, the experiment was designed using the Orthogonal Test Design Assistant software. Nine sets of experiments were performed by setting three different levels of three factors: (A) laser pulse energy, (B) the number of laser pulses used to process the sample, and (C) repetition rate of laser pulses. Each set of experiments was repeated 6 times. In the orthogonal experiment, the pulse energy of (A) was 3 mJ, 4 mJ, and 5 mJ; the number of laser processing was 400 times, 500 times, and 600 times; and the repetition rate was 100 Hz, 200 Hz, and 300 Hz. The values and the labels of the three factors and three levels is shown in Table 2. This experiment is a 3-factor, 3-level experiment, so the type of orthogonal table is $L_{9}\;\left(3^{3}\right)$, as shown in Table 3.

### 2.4. Electrochemical Impedance Measurement and Analysis

The electrochemical impedance, as a key factor for neural electrodes, is very important for neural recording and electrical stimulation, which is the main function of neural electrodes. The electrochemical measurements were taken in the saline (0.9% NaCl) at room temperature with a standard three-electrode system, using an Ag/AgCl reference electrode and a titanium (Ti) wire (1 mm in diameter) counter electrode. Electrochemical impedance spectroscopy (EIS) measurements were performed by using a voltage sinewave of 10 mV amplitude, within the frequency range from 1 Hz to 100 kHz. We optimized the electrochemical impedance by combining laser processing parameters, which is the evaluation quality index of laser processing performance on the electrode. Neural electrodes were connected to a 4 mm banana adaptor plate by a reverse flexible printed circuit (FPC).

The EIS data were analyzed and the equivalent circuit models were also built and fitted by the ZView software (North Carolina, 3.10 Version). Electrochemical impedance at the representative frequencies were analyzed to help construct equivalent circuit models. The impedance at 1 kHz is widely referred to evaluate neural electrode. In addition, the impedance of electrodes at high frequency (100 kHz) describes the characteristics of capacitance components, while the impedance at low frequency (1 Hz) describes the characteristics of other components, such as resistance.

## 3. Results and Discussion

### 3.1. Orthogonal Experimental Analysis of EIS

#### 3.1.1. Range Analysis

Range analysis is also called intuitive analysis. By calculating the range of each factor, we can find the main factors and secondary factors that affect the experimental results; that is, we can arrange the order of the factors affecting the indicator. The greater the extreme difference, the greater the influence of each factor on the experimental results. We analyze the average of the results of six replicated experiments for each set of experiments. The calculation of the range of the three factors of the nine experimental groups is shown in Table 4 and Table 5. Based on the impedance and the size of the presented design, it is too big for neural spike detection. The proposed soft neural electrode based on FPC technology and PDMS can be used for neural recording of ECoG and neural stimulation of the cerebral cortex or spinal cord in larger animal models, such as pigs and monkeys.

By optimizing the range of electrochemical impedances, the main factors affecting electrode performance at different frequencies are found. At 1 kHz, impedance is widely used to evaluate neural electrodes, and in the 200 * 450 μm rectangular template, factor A (pulse energy) > factor B (number) > factor C (repetition rate). That is, the order of factors affecting the impedance of the laser processing electrode is A > B> C. In the 200-diameter circular template, factor A (pulse energy) > factor C (repetition rate) > factor B (number). That is, the order of factors affecting the impedance of the laser processing electrode is A > C > B. Moreover, the difference between factor A (pulse energy) is significantly greater than the difference between factor B (number) and factor C (repetition rate), indicating that laser pulse energy are the main factors affecting the impedance of the electrode. Further demonstrating the phenomenon shown in Table 1, when the laser pulse energy is small, even if the number of laser strikes is large, the edges of the pattern are also not sharp. It makes sense to find the optimal laser processing parameters to precisely remove the PDMS encapsulation layer by optimizing the electrochemical impedance.

#### 3.1.2. Mean Analysis

**Figure 2:**
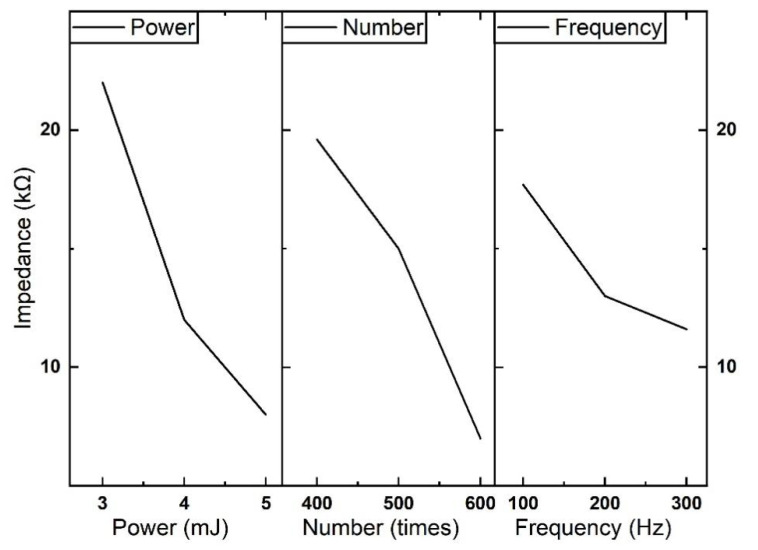
Effect curve of electrochemical impedance using 200 * 450 μm rectangular template at 1 kHz.

**Figure 3:**
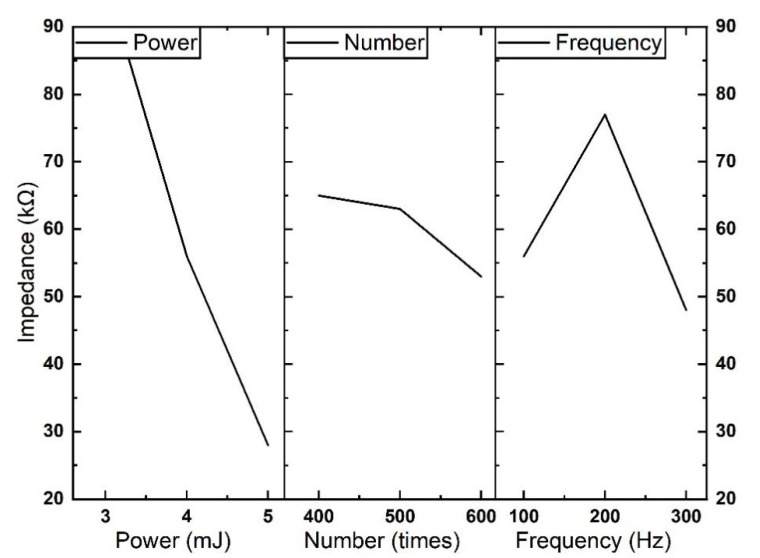
Effect curve of electrochemical impedance using 200 μm circle template at 1 kHz.

The mean calculations of the nine group experiments are shown in Table 6 and Table 7. We analyze the average of the results of six replicated experiments for each set of experiments. By optimizing the mean of the electrochemical impedance, the optimal combinations of parameters at different frequencies are found. As shown in Table 6 and Table 7, for the two templates, the best combination parameter is A3B3C3, in which the pulse energy is 5 mJ, the number is 600 times, and the repetition rate is 300 Hz at 1 kHz. Figure 2 and Figure 3 present the mean results of different factors at their respective levels in orthogonal experiments in a more intuitive way, which can further validate the conclusions of the above optimal combination of parameters. Under the same template, it is observed that the higher the laser pulse energy, the smaller the electrode impedance. Meanwhile, it is that the greater the number of laser hits, the smaller the impedance of the electrode.

At low frequencies, the characteristics of the resistance, impedance, and phase angle are described as having a strong correlation with the electrode area. At high frequencies, the characteristics of the capacitor, impedance, and phase angle are described independent of the electrode area [49,50,51]. As shown in Table 8, the total average impedance at low frequencies is significantly greater than at high frequencies in the two templates. That is, the total average at 1 Hz and 100 Hz are significantly higher than the averages at 10 kHz and 100 kHz. The experimental results obtained are consistent with the theory.

### 3.2. Orthogonal Experimental Analysis Based on Equivalent Circuit Model

By fitting the experimental electrochemical impedance data to an appropriate equivalent circuit model, the evolution of the characteristics of the electrode system in vitro can be further understood. The first step in this process requires the design and selection of a sufficient circuit model that can satisfactorily fit the data while achieving a physical interpretation of the parameters.

#### 3.2.1. Construction of Equivalent Circuit Model

**Figure 4:**
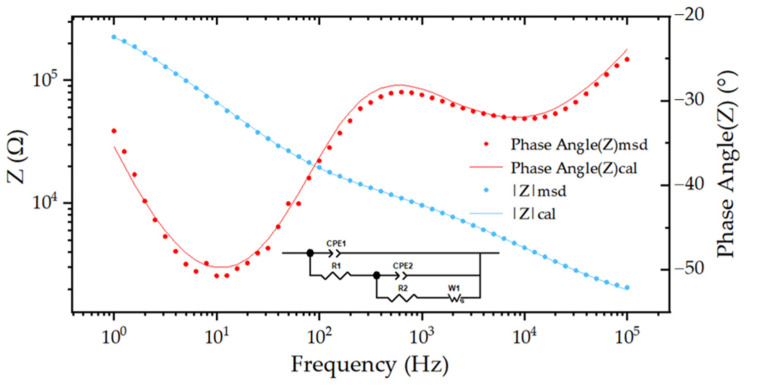
The equivalent circuit model and representative electrochemical impedance spectroscopy (EIS) of the soft neural electrode based on FPC technology and PDMS encapsulation layer. The symbols are measured EIS data, and the solid lines are fitted data by calculation. The inserted diagram is the corresponding equivalent circuit model.

By adding a non-ideal capacitor in parallel to the modified Randles circuit, the equivalent circuit model established is shown as the inserted diagram in Figure 4. Five different components describe this equivalent circuit. R1 is the pure resistance of the electrode, including the Cu wire, NaCl solution, the wire of the electrode, etc. CPE2 characterizes the non-ideal capacitive nature of the electrode interface as a constant phase element. R2 is the charge transfer resistance. The Warburg element (W1) states that the interface is mixed controlled by the charge transfer and diffusion process. CPE1 is a non-ideal parasitic capacitance which is used to consider the capacitive characteristics of the polyimide and PDMS layer. The role of the parasitic capacitor is not very prominent at low frequencies, but at high frequencies, its equivalent value increases. As displayed in Figure 4, the equivalent circuit model provides fairly good fitting to the measured EIS data. An electrochemical interface was created by the selective removal of PDMS soft encapsulation layer. Implantable neural electrodes do not reach the 100 kHz frequency when actually performing neural recording. However, it should be noticed that the capacitance of the neural electrode may lead to the leakage of stimulation current. It increases the necessity of building the equivalent circuit model and giving a detailed analysis of each component.

#### 3.2.2. Range Analysis

As shown in Table 9 and Table 10, respectively, at 1 kHz, the rectangular template of 200 * 450 μm and the circular template of 200 μm diameter fit the data for each component of the equivalent circuit model. By optimizing the difference in the electrochemical impedance, the dominant factors affecting the fitting effect of each component are found. As shown in Table 9, in the rectangular template of 200 * 450 μm, for R2 (charge transfer resistance) and R1 (resistance of the solution), factor A (pulse energy) > B (number) > factor C (repetition rate); that is, the order of laser processing factors that affect the impedance of the electrode is A > B > C. For CPE1 (parasitic capacitance), CPE2 (characterizing non-ideal constant phase elements), and Warburg components, factor B (number) is the dominant factor. As shown in Table 10, for R1 (solution resistance), R2 (charge transfer resistance), and Warburg components, factor A (pulse energy) is the dominant factor.

As shown in Table 9, for R2 (charge transfer resistance) at 200 * 450 μm rectangular template, factor A (pulse energy) > B (number) > factor C (repetition rate). This phenomenon is the same as the dominant factor analysis results of the two templates in Table 4 at 1 kHz, which further proves the reliability of the experimental results.

#### 3.2.3. Mean Analysis

The total optimal combinations of each component parameters are shown in Table 11 and Table 12. Based on different components or their parameters, the optimal combinations vary from each other, which can contribute to revealing the detailed effect of the laser micro-machining of PDMS as an encapsulation layer. The optimal combinations of laser parameters based on the equivalent circuit model are different from that of EIS. It should be also noted that size of the electrode also impacts the combination of laser parameters. For R2 as the charge transfer resistance, the 200 * 450 μm rectangular size requires more pulse energy than that of the 200-diameter circular size. In the equivalent circuit model, the smaller the Warburg element, the better it will behave as a neural interface. Here, we focus on the discussion of W-R as the diffusion resistance. By optimizing the mean of the electrochemical impedance of each fitted component, the combinations of parameters that can achieve the best fit are found. As shown in Table 11, for W at the 200 * 450 μm rectangular template fitted, the optimal combination of parameters is A3B3C2; that is, the pulse energy is 5 mJ, the number is 600 times, and the repetition rate is 200 Hz. Moreover, for W at the 200-diameter circular template fitted, the optimal combination of parameters is A3B3C3; that is, the pulse energy is 5 mJ, the number is 600 times, and the repetition rate is 300 Hz.

PDMS has important application value in the areas of implants, including the neural interfaces, yet selective removal of the PDMS encapsulation layer is still a big challenge. In the paper, we proposed and successfully prepared a soft electrode based on the FPC technology and PDMS encapsulation layer by using an excimer laser as a cold micromachining tool. The influence of orthogonal laser cutting parameters on the electrochemical impedance of neural electrodes was investigated and optimized. Our experimental results show that excimer lasers can be applied in the micromachining of implantable neural interfaces, which can remove the PDMS with high selectivity and cause no damage to the other materials of neural electrode. Furthermore, The Young’s modulus of flexible electrode that is made of polyimide or parylene is in the order of GPa (109 Pa). Meanwhile, PDMS is so intrinsically soft that its Young’s modulus is in the order of MPa (106 Pa). Using the soft PDMS layer to encapsulate the flexible electrode will improve the mechanical contact between the soft neural tissue and flexible electrode materials, which has the potential to reduce the mechanically induced foreign body reaction. It should also be mentioned that the Young’s modulus of rigid electrode that is made of silicon or metal is in the order of hundreds GPa (1011 Pa), and the rigid electrode could also benefit from this approach.

## 4. Conclusions

We demonstrated the soft neural electrode based on FPC technology and a PDMS encapsulation layer. Using an excimer laser as a cold micro-machining tool, the PDMS encapsulation layer that exposes the electrode sites in the implantable neural interface is precisely removed. The effect of the laser cutting parameters on the electrochemical impedance of neural electrodes was studied and optimized. The analysis on electrode impedance data at different frequencies helps to find the optimal combination of excimer laser cutting parameters, including range analysis and mean analysis. In addition, the optimal combinations of laser parameters based on the equivalent circuit model are different from that of EIS, which can reveal the details of the electrochemical properties of neural electrodes with PDMS as an encapsulation layer. Suitable laser cutting parameters allow the precise cutting of the PDMS of implantable neural electrodes without compromising electrode performance. Combined with the softness and biocompatibility of PDMS, this cold micromachining approach is expected to improve the soft mechanical properties of flexible and rigid implantable neural electrodes.

## Figures and Tables

**Table 1:** Combinations of parameters too high or too low lead to unsatisfactory experimental results.

	Low Pulse Energy (<3 mJ)	High Pulse Energy (>5 mJ)
small number (<400 times)	PDMS is not penetrated	electrode is damaged
large number (>600 times)	PDMS cutting edges are not neat	electrode is damaged

**Table 2:** The values and the labels of the three factors and three levels. For example, 3 mJ is labeled as A1.

Level	(A) Pulse Energy (mJ)	(B) Number (Times)	(C) Repetition Rate (Hz)
1	3	400	100
2	4	500	200
3	5	600	300

**Table 3:** Nine sets of laser parameter combinations. The combinations are: A1B1C1, A1B2C2, A1B3C3, A2B1C2, A2B2C3, A2B3C1, A3B1C3, A3B2C1, and A3B3C2.

Experimental Label	(A) Pulse Energy (mJ)	(B) Number (Times)	(C) Repetition Rate (Hz)
1	3	400	100
2	3	500	200
3	3	600	300
4	4	400	200
5	4	500	300
6	4	600	100
7	5	400	300
8	5	500	100
9	5	600	200

**Table 4:** The range analysis data for 200 * 450 μm rectangular template.

200 * 450 μm	1 Hz (Ω)	100 Hz (Ω)	1 KHz (Ω)	10 KHz (Ω)	100 KHz (Ω)
(A) Pulse energy	51,247.3	25,884.9	14,031.7	7414.3	2230.9
(B) Number	50,614.4	20,682.5	12,419.0	6670.6	2506.1
(C) Repetition rate	34,187.2	10,744.1	6065.7	3012.6	609.6
Dominant factor	A > B > C	A > B > C	A > B > C	A > B > C	B > A > C

**Table 5:** The range analysis data for 200-diameter circular template.

200 μm	1 Hz (Ω)	100 Hz (Ω)	1 KHz (Ω)	10 KHz (Ω)	100 KHz (Ω)
(A) Pulse energy	338,858.1	165,371.1	69,091.4	29,252.6	9197.4
(B) Number	71,222.3	33,870.8	12,170.4	5193.7	2462.5
(C) Repetition rate	140,860.4	63,946.0	29,342.0	11,636.4	4932.8
Dominant factor	A > C > B	A > C > B	A > C > B	A > C > B	A > C > B

**Table 6:** Mean value data for 200 * 450 μm rectangular template.

200 * 450 μm	1 Hz (Ω)	100 Hz (Ω)	1 KHz (Ω)	10 KHz (Ω)	100 KHz (Ω)
(A1) 3 mJ	89,721.5	41,472.2	22,061.0	11,808.7	4986.2
(A2) 4 mJ	58,563.3	24,742.0	12,327.4	5561.9	2755.3
(A3) 5 mJ	38,474.2	15,587.3	8029.3	4394.4	2809.0
(B1) 400 times	87,945.6	36,177.0	19,685.7	10,300.0	4647.5
(B2) 500 times	61,482.2	30,129.9	15,465.4	7835.6	3761.4
(B3) 600 times	37,331.2	15,494.5	7266.7	3629.4	2141.5
(C1) 100 Hz	83,848.9	33,829.2	17,694.1	8859.4	3818.6
(C2) 200 Hz	49,661.7	24,887.2	13,095.2	7058.7	3522.8
(C3) 300 Hz	53,248.3	23,085.1	11,628.4	5846.9	3209.0
Best combination	A3B3C2	A3B3C3	A3B3C3	A3B3C3	A2B3C3

**Table 7:** Mean value data for 200-diameter circular template.

200 μm	1 Hz (Ω)	100 Hz (Ω)	1 KHz (Ω)	10 KHz (Ω)	100 KHz (Ω)
(A1) 3 mJ	479,222.6	231,745.8	97,450.9	39,149.3	12,563.4
(A2) 4 mJ	232,688.2	144,667.0	56,782.2	16,139.2	4769.2
(A3) 5 mJ	140,364.5	66,374.7	28,359.5	9896.7	3366.0
(B1) 400 times	279,028.0	168,608.7	65,751.1	20,443.0	5756.0
(B2) 500 times	322,234.8	139,440.8	63,260.8	24,967.9	8218.4
(B3) 600 times	251,012.5	134,737.9	53,580.7	19,774.3	6724.2
(C1) 100 Hz	255,215.3	143,257.6	56,096.1	16,784.8	4376.9
(C2) 200 Hz	368,960.2	181,737.9	77,919.2	28,421.2	9309.7
(C3) 300 Hz	228,099.8	117,792.0	48,577.3	19,979.1	7011.9
Best combination	A3B3C3	A3B3C3	A3B3C3	A3B3C1	A3B1C1

**Table 8:** Total average data for the corresponding frequency.

	1 Hz (Ω)	100 Hz (Ω)	10 kHz (Ω)	100 kHz (Ω)
Total average	173,172.4	87,431.5	14,491.7	5208.2

**Table 9:** The range analysis data for 200 * 450 μm rectangular template from the key components of equivalent circuit model.

200 * 450 μm	R1(×10^4^) (Ω)	R2 (×10^4^) (Ω)	CPE1 (×10^−10^) (F)	CPE2 (×10^−8^) (F)	W (×10^5^) (F)
(A) Pulse energy	0.72	2.53	1.13	1.98	0.60
(B) Number	0.63	1.90	1.14	2.97	0.94
(C) Repetition rate	0.18	0.80	0.27	1.43	0.78
Dominant factor	A > B > C	A > B > C	B > A > C	B > A > C	B> A > C

**Table 10:** The range analysis data for 200-diameter circular template from the key components of equivalent circuit model.

200 μm	R1 (×10^4^) (Ω)	R2(×10^4^) (Ω)	CPE1 (×10^−10^) (F)	CPE2 (×10^−9^) (F)	W (×10^5^) (F)
(A) Pulse energy	4.18	22.43	2.10	2.97	3.27
(B) Number	0.20	3.07	0.94	3.50	1.50
(C) Repetition rate	1.02	5.13	3.31	1.43	2.07
Dominant factor	A > B > C	A > C > B	C > A > B	B > A > C	A > C > B

**Table 11:** Mean analysis data for 200 * 450 μm rectangular template from the key components of equivalent circuit model.

200 * 450 μm	R1 (×10^4^) (Ω)	R2 (×10^4^) (Ω)	CPE1 (×10^−10^) (F)	CPE2 (×10^−8^) (F)	W (×10^5^) (F)
(A1) 3 mJ	1.10	3.43	3.33	3.43	1.39
(A2) 4 mJ	0.53	2.40	3.83	1.45	1.13
(A3) 5 mJ	0.38	0.90	2.70	3.23	0.79
(B1) 400 times	0.93	2.37	2.63	1.53	1.63
(B2) 500 times	0.78	3.13	3.47	2.09	0.99
(B3) 600 times	0.30	1.23	3.77	4.50	0.69
(C1) 100 Hz	0.78	2.33	3.17	1.93	1.57
(C2) 200 Hz	0.63	1.80	3.27	3.37	0.79
(C3) 300 Hz	0.60	2.60	3.43	2.82	0.96
Best combination	A3B3C3	A3B3C2	A3B1C1	A2B1C1	A3B3C2

**Table 12:** Mean analysis data for 200-diameter circular template from the key components of equivalent circuit model.

200 μm	R1 (×10^4^) (Ω)	R2(×10^4^) (Ω)	CPE1 (×10^−10^) (F)	CPE2 (×10^−9^) (F)	W (×10^5^) (F)
(A1) 3 mJ	5.47	28.37	2.46	6.47	5.77
(A2) 4 mJ	2.30	14.13	4.55	7.77	3.17
(A3) 5 mJ	1.28	5.93	4.30	9.43	2.50
(B1) 400 times	2.93	17.77	4.30	6.40	3.47
(B2) 500 times	3.13	14.70	3.36	7.37	4.73
(B3) 600 times	2.98	15.97	3.66	9.90	3.23
(C1) 100 Hz	2.83	15.03	5.66	7.33	3.57
(C2) 200 Hz	3.62	19.27	2.35	7.57	4.97
(C3) 300 Hz	2.60	14.13	3.30	8.77	2.90
Best combination	A3B1C3	A3B2C3	A1B2C2	A1B1C1	A3B3C3

## Data Availability

The data presented in this study are available on request from the corresponding author.
